# Peak CK-MB has a strong association with chronic scar size and wall motion abnormalities after revascularized non-transmural myocardial infarction – a prospective CMR study

**DOI:** 10.1186/s12872-018-0767-7

**Published:** 2018-02-08

**Authors:** Pauli Pöyhönen, Minna Kylmälä, Paula Vesterinen, Sari Kivistö, Miia Holmström, Kirsi Lauerma, Heikki Väänänen, Lauri Toivonen, Helena Hänninen

**Affiliations:** 1 Heart and Lung Center, Helsinki University Hospital and Helsinki University, Haartmaninkatu 4, 00029 HUS, Po BOX 340, Helsinki, Finland; 2 HUS Medical Imaging Center, Radiology, Helsinki University Hospital, Helsinki, Finland; 3 Department of Biomedical Engineering and Computational Science, Aalto University, Espoo, Finland

**Keywords:** Coronary artery disease, Acute myocardial infarction, Infarct transmurality, Left ventricular remodeling, Cardiovascular magnetic resonance, Creatine kinase-MB

## Abstract

**Background:**

Large myocardial infarction (MI) is associated with adverse left ventricular (LV) remodeling (LVR). We studied the nature of LVR, with specific attention to non-transmural MIs, and the association of peak CK-MB with recovery and chronic phase scar size and LVR.

**Methods:**

Altogether 41 patients underwent prospectively repeated cardiovascular magnetic resonance at a median of 22 (interquartile range 9–29) days and 10 (8–16) months after the first revascularized MI. Transmural MI was defined as ≥75% enhancement in at least one myocardial segment.

**Results:**

Peak CK-MB was 86 (40–216) μg/L in median, while recovery and chronic phase scar size were 13 (3–23) % and 8 (2–19) %. Altogether 33 patients (81%) had a non-transmural MI. Peak CK-MB had a strong correlation with recovery and chronic scar size (*r* ≥ 0.80 for all, *r* ≥ 0.74 for non-transmural MIs; *p* < 0.001). Peak CK-MB, recovery scar size, and chronic scar size, were all strongly correlated with chronic wall motion abnormality index (WMAi) (*r* ≥ 0.75 for all, *r* ≥ 0.73 for non-transmural MIs; *p* < 0.001). There was proportional scar size and LV mass resorption of 26% (0–50%) and 6% (− 2–14%) in median. Young age (< 60 years, median) was associated with greater LV mass resorption (median 9%vs.1%, *p* = 0.007).

**Conclusions:**

Peak CK-MB has a strong association with chronic scar size and wall motion abnormalities after revascularized non-transmural MI. Considerable infarct resorption happens after the first-month recovery phase. LV mass resorption is related to age, being more common in younger patients.

## Background

Left ventricular (LV) remodeling after acute myocardial infarction (MI) is a major precursor of heart failure [1, 2] and predictor of mortality [3]. Remodeling occurs despite successful reperfusion and sustained patency of the infarct-related artery [4]. Early remodeling during first weeks after MI involves infarct expansion and scar formation, while late remodeling involves adaptive changes in the non-infarcted myocardium, such as myocyte hypertrophy, chamber dilatation or interstitial fibrosis, to balance increased loading conditions [5, 6].

The best predictor of LV dysfunction after MI is the infarct size [2, 7]. Most past studies have focused on large MIs and subsequent global LV remodeling, and it has been presented that remodeling rarely occurs with infarct size less than 18.5% of LV volume [8]. However, due to improved revascularization therapy, chronic infarct size tends to be smaller. The course of LV remodeling after smaller non-transmural MIs is less well established and there is still a lack of prospective studies with repeated comprehensive cardiovascular magnetic resonance (CMR) imaging. CMR provides high fidelity and normalized information of the LV anatomy, global and local function, and tissue injury after MI [9], which enables also the detection of subtle changes. The preferred biomarkers of cardiac necrosis after MI are troponins due to high clinical sensitivity and myocardial tissue specificity, and CK-MB is the best alternative [10]. Troponins and CK-MB have all showed good correlation with infarct size and global ejection fraction after large reperfused MI, although the relation between biomarkers and infarct size might be less robust in smaller infarcts [11].

The purpose of this study was therefore, using repeated CMR imaging, to study 1) the course of LV remodeling after the first revascularized spontaneous acute MI, 2) the more subtle remodeling process after non-transmural MI, and 3) the association of peak CK-MB with recovery phase (1–4 weeks) and chronic phase (> 6 months) scar size, and global and local LV dysfunction. We also made a post-hoc analysis of long-term survival.

## Methods

### Patients and study design

This is a CMR substudy of the prospectively conducted ISKE project (Imaging acute myocardial ischemia by body surface potential mapping) [12]. In brief, from June 2003 to June 2005, patients admitted to the Coronary Care Unit of Helsinki University Hospital for suspected acute coronary syndrome were screened during office hours. Inclusion criteria were prolonged chest pain ≥20 min within 48 h of recruitment, associated with evidence of acute ischemia in the initial 12-lead electrocardiogram (ST-segment elevation, −depression, or T-wave inversion in ≥2 contiguous leads), or elevated cardiac enzymes (CK-MB mass > 7 μg/L or troponin T > 0.03 μg/L), or both [12]. Patients with bundle branch block, atrial fibrillation, pacemaker, or need for ventilator support, were excluded. Also patients with contraindications for CMR, such as pacemakers, defibrillators or claustrophobia, were excluded.

In this CMR substudy, all eligible patients were diagnosed with acute spontaneous type 1 MI [10], with elevated cardiac enzymes or scar at CMR, and underwent revascularization in the acute phase. Both patients with ST-elevation MI (STEMI) and non-ST elevation MI (NSTEMI) at presentation were included. All patients with prior MI, percutaneous coronary intervention (PCI), or coronary artery bypass craft (CABG), were excluded.

All patients were treated with successful PCI, CABG or thrombolysis in less than 3 days after the admission except for one patient treated successfully with CABG after 9 days during the same hospital stay, and received optimal secondary preventive medication for coronary artery disease.

All patients underwent two CMR examinations after MI to assess LV and infarct remodeling. The first CMR study occurred during the recovery phase 7–30 days after MI, and the second at the chronic infarct phase ≥6 months after MI. Revascularization was always performed before the first CMR.

CK-MB was measured according to routine clinical praxis: at arrival at the hospital, the following evening or morning, or both. The maximum value of these measurements, peak CK-MB, served as a measure of infarct size. In most patients, measurement of peak CK-MB occurred 12–24 h after the onset of chest pain.

### CMR methods

CMR imaging was performed with a 1.5-T imager (Sonata, Siemens Medical Solutions) using body-array coil as a receiver. Breath-hold cine CMR was performed using retrospectively electrocardiographically gated segmented imaging with steady-state free-precession (SSFP). Cine CMR images were acquired in vertical, horizontal long-axis and short-axis planes covering the whole LV. Typical imaging parameters were TR/TE 3.0/1.51 ms, flip angle 52 degrees, 256 × 256 matrix and 240 × 340 mm field of view. Slice thickness was 6 mm. The temporal resolution was 42–47 ms.

Ten to fifteen minutes after intravenous injection of a contrast agent (gadodiamide, Omniscan TM, GE Healthcare, 0.2 mmol/kg), late gadolinium enhancement (LGE) images were acquired in the same views as for cine images, using inversion-recovery turbo fast-low angle shot (FLASH). Typical imaging parameters were TR/TE 8.6/4.3 ms, 256 × 256 matrix, slice thickness 8 mm and interslice gap 20%. Inversion times were optimized to null the signal intensity of normal myocardium (250–300 ms).

### CMR analysis

CMR images were analyzed by two experienced observers blinded to clinical outcome. LV mass, end-diastolic (EDV) and end-systolic (ESV) volumes were evaluated by manually tracing the epicardial border (excluding epicardial fat) and endocardial borders (excluding papillary muscles) at end-diastole and -systole for short-axis slices. Volumetric indices were obtained by dividing values by body surface area.

LGE images were analyzed using the standard 17-segment model of the LV, leaving out the apex [13]. Scar area was manually delineated, the method of which has recently shown the lowest overall variability for quantification of MI when analyzed by experienced observers [14]. The global scar size was calculated by dividing the sum of segmental scar areas by the total area of all segments. Infarct transmurality was evaluated as the percentage area in each segment. Transmural MI was defined as ≥75% enhancement in at least one myocardial segment on recovery phase CMR [15], irrespectively of STEMI/NSTEMI classification at presentation.

Repeatability of LGE analyses was also studied: 384 segments were analyzed for the presence of scar by two independent observers, and results were concordant for 366 (95%) of the segments (kappa = 0.86).

Segmental wall motion was visually estimated, blinded to LGE results. The degree of wall motion abnormality in each segment was scored as 0 (normokinesia), 1 (hypokinesia), 2 (akinesia) or 3 (dyskinesia). Wall motion abnormality index (WMAi) of the LV was then calculated as the sum of all segmental scores.

### Follow-up

Follow-up data of major adverse cardiac events (MACEs) was post-hoc collected until the end of year 2012 based on local and national registries, including university and local hospital records, general practitioners’ records and the mortality information from the public organization Statistics Finland, without direct contact to patients. The survival time was calculated from the first CMR and only one event was tabulated per subject. A MACE was defined as cardiovascular death, aborted sudden death, heart failure hospitalization, or recurrent MI. Aborted sudden death was defined as documented resuscitation from cardiac arrest or appropriate implantable cardioverter defibrillator therapy, i.e. antitachycardia pacing or shock, for ventricular tachycardia or fibrillation.

### Statistical analysis

Continuous variables are presented as median (interquartile range [IQR]) and categorical variables as frequency (%). Comparison between continuous variables was performed with Mann-Whitney U test. CMR variables were compared between recovery and chronic phase studies using related samples Wilcoxon signed rank test. Correlation between variables was calculated using Spearman’s method. Linear regression was performed to identify predictors of chronic phase ejection fraction (EF), reaching sufficient normality of residuals. In survival analysis, Cox regression analysis was performed to identify predictors of MACEs. A *p*-value of < 0.05 was considered statistically significant and all tests were 2-sided. Statistical analysis was performed on SPSS 21 statistical package (SPSS, Chigaco, IL).

## Results

### Study group and baseline data

**Fig. 1:**
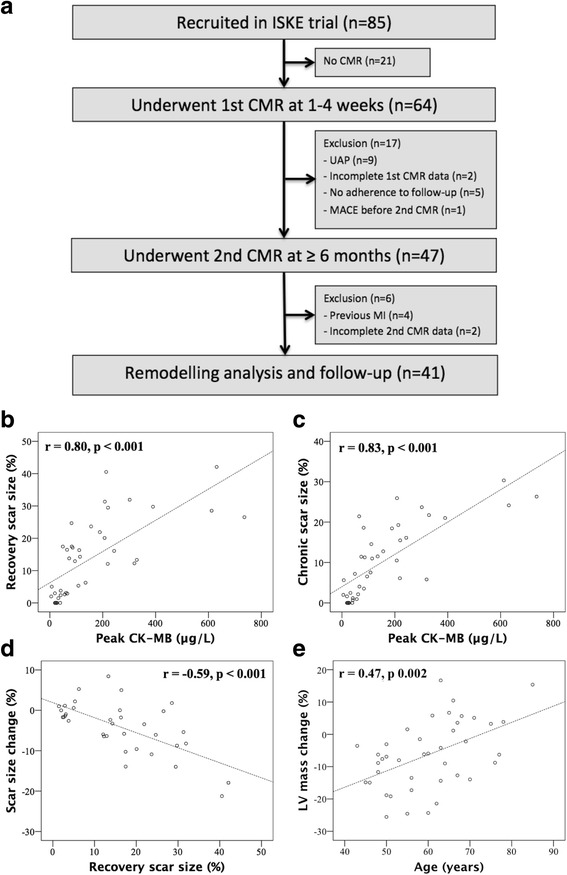
Study flow chart (**a**). Correlation of peak CK-MB with recovery (**b**) and chronic (**c**) scar size at CMR (*n* = 41). Correlation of recovery scar size with scar size change between CMRs (**d**); only patients with visible scar (*n* = 35) included. Correlation of age with left ventricular (LV) mass change between CMRs (**e**) (*n* = 41). Abbreviations: CMR cardiovascular magnetic resonance, MI myocardial infarction, UAP unstable angina pectoris, MACE major adverse cardiac event

From June 2003 to June 2005, altogether 85 patients were enrolled as a part of the ISKE project. Derivation of the CMR study group of 41 patients with their first revascularized MI and complete CMRs data are presented in Fig. 1A. Patients presented with a median peak CK-MB of 86 μg/L (range 5–736 μg/L, IQR 40–216 μg/L) (Table 1). Half (47%) of the patients had a multiple vessel disease and 59% anterior infarct. Thirty-five (85%) patients were treated with PCI, four (10%) with CABG and 2 (5%) with thrombolysis only.

**Table 1:** Baseline characteristics (*n* = 41)

Demographics	
Age, year	60 (50–67)
Gender, male	34 (83)
Body mass index, kg/m2	27 (24–30)
Cardiovascular risk factors	
Dyslipidemia	35 (85)
Hypertension	14 (34)
Diabetes	8 (20)
Smoking	16 (39)
Family risk for coronary artery disease	25 (61)
ST-elevation myocardial infarction	33 (81)
Culprit coronary artery	
Left anterior descending (or left main)	24 (59)
Circumflex artery	5 (12)
Right coronary	12 (29)
Multiple vessel disease	19 (46)
Anterior infarct	24 (59)
Method of reperfusion	
Thrombolysis	2 (5)
Primary percutaneus coronary intervention	35 (85)
Coronary artery bypass graft	4 (10)
Peak CK-MB (μg/L)	86 (40–216)
Discharge medication	
Aspirin	38 (93)
Clopidogrel	37 (90)
Beta blocker	40 (98)
ACE-inhibitor/AT2-blocker	26 (63)
Statin	40 (98)
Diuretic	3 (7)
Nitrate	1 (2)
Warfarin	2 (5)

Values are median (interquartile range) or n (%)

### Recovery phase CMR study

Recovery phase CMR study was performed in a median of 22 (9–29) days after hospital admission. Median scar size was 13% (range 0–42%, IQR 3–23%) of the LV (Table 2). Six (15%) patients with minor infarcts (peak CK-MB range 19–39 μg/L) had no visible scar at CMR, while two patients with small scars (2 and 5%) had normal peak CK-MB (5 and 7 μg/L). Altogether 33 patients (81%) had a non-transmural MI with a median peak CK-MB of 66 (30–146) μg/L, while eight had a transmural MI with a median peak CK-MB of 261 (209–557) μg/L.

**Table 2:** Left ventricular remodeling: comparison of CMR indices (*n* = 41)

	Recovery phase	Chronic phase	*p*-value
LV end-diastolic volume, ml/m2	79 (61–90)	70 (65–82)	0.115
LV end-systolic volume, ml/m2	39 (28–48)	36 (28–43)	0.197
Stroke volume, ml/m2	38 (33–44)	37 (31–42)	0.138
LV ejection fraction, %	52 (45–57)	51 (45–56)	0.791
LV mass, g/m2	78 (69–84)	72 (61–82)	0.001
WMAi, score	6 (3–12)	5 (1–9)	< 0.001
Scar size, % of LV	13 (3–23)	8 (2–19)	0.001

Values are median (interquartile range)

*Abbreviations: CMR* cardiovascular magnetic resonance, *LV* left ventricular. *WMAi* Wall motion abnormality index

In the whole study group (*n* = 41), peak CK-MB had a strong correlation with recovery phase scar size (*r* = 0.80, *p* < 0.001) (Fig. 1B), moderate correlation with ESV, EF and WMAi, weak correlation with EDV, and showed a trend with LV mass (*r* = 0.285, *p* = 0.071) (Table 3). There were similar correlations in the subgroup of non-transmural MIs.

**Table 3:** Correlation between peak CK-MB and scar size, left ventricular volumes, ejection fraction (EF) and wall motion abnormality index (WMAi) at recovery and chronic phase after myocardial infarction

	Recovery phase CMR (correlation to peak CK-MB)		Chronic phase CMR (correlation to peak CK-MB)	
	All (*n* = 41)	Non-transmural MI (*n* = 33)	All (*n* = 41)	Non-transmural MI (*n* = 33)
Scar size, %	0.80 (*p* < 0.001)	0.74 (*p* < 0.001)	0.83 (*p* < 0.001)	0.78 (*p* < 0.001)
EDV, ml/m2	0.49 (*p* = 0.001)	0.47 (*p* = 0.006)	0.31 (*p* = 0.050)	0.317 (*p* = 0.072)
ESV, ml/m2	0.66 (*p* < 0.001)	0.56 (*p* = 0.001)	0.53 (*p* < 0.001)	0.42 (*p* = 0.015)
EF, %	−0.64 (*p* < 0.001)	−0.52 (*p* = 0.002)	−0.62 (*p* < 0.001)	0.45 (*p* = 0.008)
WMAi, score	0.69 (*p* < 0.001)	0.60 (*p* < 0.001)	0.75 (*p* < 0.001)	0.73 (*p* < 0.001)

Values are Spearman correlation coefficients (*p*-value)

*Abbreviations: CMR* cardiovascular magnetic resonance, *EDV* end-diastolic volume, *EF* ejection fraction, *ESV* end-systolic volume, *WMAi* wall motion abnormality index

### Chronic phase CMR study

Chronic phase CMR study was performed in a median of 10 (8–16) months after hospital admission.

Peak CK-MB had a strong correlation with chronic scar size (*r* = 0.83, *p* < 0.001) (Fig. 1C) and chronic WMAi (*r* = 0.75, *p* < 0.001) (Table 3), moderate correlation with chronic ESV and EF, and showed a trend with EDV, but not with chronic LV mass (*r* = 0.22, *p* = 0.175). Similar, but weaker correlations with ESV and EF, were found in non-transmural MI group.

**Fig. 2:**
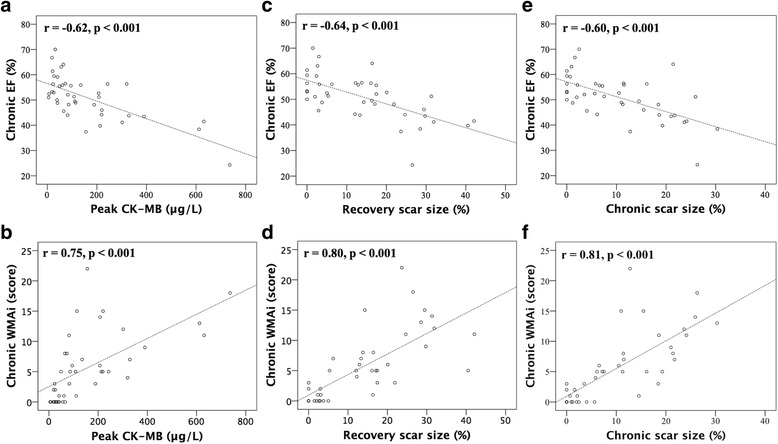
Correlation of peak CK-MB (**a**, **b**), recovery scar size (**c**, **d**) and chronic scar size (**e**, **f**) with chronic ejection fraction (EF) and wall motion abnormality index (WMAi) (*n* = 41)

Also, both recovery and chronic phase scar size had a strong correlation with chronic WMAi (r ≥ 0.80, *p* < 0.001 for both) (Fig. 2), and similar strong correlations were found in non-transmural MI group.

### Late remodeling

Between CMRs, there were no changes in median volumes or EFs, but there was a significant reduction in median scar size (13 vs. 8%, *p* = 0.001), LV mass (78 g/m2 vs. 72 g/m2, *p* = 0.001), and WMAi (6 vs. 5, *p* < 0.001) (Table 2).

Peak CK-MB or recovery scar size were not associated with late remodeling, i.e. change in LV mass or WMAi between CMRs. Considering the association of all continuous baseline and recovery phase CMR variables with late remodeling parameters, the only significant correlation was found between age and LV mass change (*r* = 0.47, *p* = 0.002), meaning that younger age was associated with greater LV mass resorption (Fig. 1E). The proportional LV mass resorption was 6% in median (from 2% increase to 14% reduction), while younger half of the patients (< 60 years) experienced median LV mass resorption of 9% (6–17%) vs. 1% (1% increase to 10% resorption) in older ones (*p* = 0.007).

In 35 patients with visible scar at CMR, the median absolute and proportional scar size resorption between CMRs were 3% (0–8%) and 26% (0–50%). Absolute scar change correlated with recovery phase scar size (*r* = − 0.59, *p* < 0.001) (Fig. 1D), i.e. large MIs had greater absolute scar resorption. However, proportional scar resorption was not associated with scar size.

### Predictors of chronic EF

Univariate predictors of chronic EF included peak CK-MB, recovery phase scar size, EDV, ESV, EF, LV mass and WMAi. By multivariate regression (stepwise forward), the significant predictors of chronic phase EF were recovery EF (Beta = 0.595, *p* < 0.001) and peak CK-MB (Beta = − 0.312, *p* = 0.012).

### Long-term survival

Nine (21%) patients reached a MACE during a median follow-up of 7.7 (4.1–8.5) years. There were one cardiovascular death, one aborted sudden death, two heart failure hospitalizations and five recurrent MIs. In univariate Cox regression analysis of all baseline, recovery and chronic phase CMR indices, and late remodelling variables, the only significant predictor of MACEs was WMAi at chronic phase (hazard ratio [HR] 1.15, 95% confidence interval [CI] 1.01–1.31, *p* = 0.038). Multiple vessel disease (HR 4.6, 95% CI 0.9–22.3, *p* = 0.060), EDV late remodelling (HR = 1.04, 95% CI 1.00–1.08, *p* = 0.051) and WMAi late remodeling (HR = 1.23, 95% CI 0.98–1.55, *p* = 0.068) showed a trend in predicting MACEs.

## Discussion

### Main findings

The main finding of this study is that peak CK-MB has a strong association with chronic scar size and WMAi after revascularized MI, with similar correlations in patients with non-transmural MIs only. Peak CK-MB predicted also chronic EF, independently of recovery phase EF. After the first weeks recovery phase, there still happens considerable proportional infarct resorption of one quarter in median, independently of infarct size, and milder reduction of LV mass and WMAi. Greater LV mass resorption was associated with younger age.

### Peak CK-MB, recovery and chronic scar size

Before the era of acute coronary reperfusion, it was shown that the peak value of serially sampled CK-MB after acute MI significantly correlates with scar size at autopsy [16]. More recently, peak CK-MB has been shown to correlate well with infarct size after reperfused MI [11, 17–19]. In these studies, patients usually had large MIs. Our finding that peak CK-MB has a strong association with recovery phase and chronic phase scar size after revascularized MI supports earlier studies, and adds to current knowledge that this association is valid also in smaller non-transmural infarcts.

In this study, CMR missed small infarcts with peak CK-MB ranging from 19 to 39 μg/L. This is in accordance with a previous study by Choi et al. [20], in which patients with peak CK-MB less than 38 μg/L after the first revascularized MI had no enhancement on CMR, and a study by Hedström et al. [17] .

In our study, only 19% of patients had a transmural MI by definition, although 81% of patients presented with STEMI. However, this is similar to a recent study by Rodriguez-Palomares JF et al. [21], in which 22% of STEMI patients treated with PCI had transmurality index more than 75% enhancement of wall thickness in acute phase CMR 15 min after contrast injection. Thus, due to early revascularization, most of the infarcts in our study did not evolve to fully transmural.

### Infarct resorption

We found that after the first weeks recovery phase, there still happens considerable proportional infarct resorption of 26% in median before chronic infarct phase. This is in accordance with studies by Pokorney et al. [22], demonstrating 32% and 12% decrease in infarct mass from 1 week to 4 months and 4 months to 14 months, and by Lund et al. [23], demonstrating 26% infarct resorption between 5 days and 8 months, after reperfused MI. As Pokorney et al., we showed that proportional infarct resorption is independent of initial infarct size.

### Infarct size and LV dysfunction

EF and ESV have been established as strong predictors of cardiac outcome after MI [24], while infarct size as the best predictor of LV dysfunction [2, 7]. We found that peak CK-MB is correlated with recovery and chronic phase ESV, EF and WMAi after revascularized MI, with similar correlations in non-transmural MIs only. In a recent study of patients with large anterior ST-elevation myocardial infarction (median peak CK-MB 240 IU/L) treated with primary PCI, peak CK-MB was associated with EF (*r* = − 0.56, *p* < 0.001) at 1 month follow-up CMR [19]. In another study of patients with large reperfused MI, all cardiac biomarkers CK-MB, troponin T, and troponin I correlated with EF at 1 month follow-up single-photon emission computer tomography [11]. Earlier, it has been presented that LV remodeling rarely occurs with infarct size less than 18.5% of LV volume [8]. In a study by Lund et al. it was shown that infarct size of 24% or more is an important predictor of remodeling (20% increase in LV end-diastolic diameter) between acute and chronic phase [23]. Our patients had mainly non-transmural infarcts, with a median peak CK-MB of 86 μg/L and scar size of 13% at 1 month CMR. Thus, we showed that also after smaller non-transmural MIs, peak CK-MB, as well as recovery and chronic scar size, correlate with chronic global and local LV dysfuntion. Furthermore, the best overall model to predict chronic EF in our whole study group included peak CK-MB and recovery phase EF.

Few prospective data is available on the association between infarct size and chronic local segmental wall motion abnormalities after revascularized acute MI. Our finding that peak CK-MB, recovery scar size, and chronic scar size, all correlate strongly with chronic WMAi (*r* ≥ 0.75, *p* < 0.001), and similarly in non-transmural MIs only (*r* ≥ 0.73, *p* < 0.001), is in accordance with a recent retrospective analysis of patients with previous (> 6 months) MI, in which infarct size correlated with WMAi but the association was more significant in transmural compared to non-transmural MIs [25] .

Large infarct size has been previously associated with progressive LV remodeling [7]. In our study, neither peak CK-MB nor scar size at recovery phase were associated with late remodeling between recovery and chronic phases. This is probably due to fact that most of our patients had small to intermediate sized infarcts and that the infarct related remodeling occurred primarily during the first weeks recovery phase. Earlier, it has been suggested that late remodeling would be triggered by factors different from initial infarct size, such as progressive ischemia [4]. The only predictor of late remodeling in our patients was age; younger half of patients having greater LV mass resorption (median 9% vs. 1%, *p* = 0.007). Left ventricular hypertrophy is an important compensatory mechanism after MI, but prolonged progression of cardiac hypertrophy leads to heart failure. It has been presented that post-infarction LV remodeling is more pronounced in elderly patients due to inability to cope with increased myocardial stress [26], explaining the association between aging heart and milder late LV mass resorption in our study.

### Predictors of cardiac survival

In our patients, chronic WMAi was the only predictor of MACEs in the long-term, while multiple vessel disease, late increase in EDV or WMAi, showed tendency toward adverse prognosis. Predictors of long-term outcome in our study may be more related to severity of coronary artery disease than infarct size, since the median scar size was relatively small and most MACEs were recurrent MIs. Earlier, both WMAi and scar size have been shown to be independent predictors of MACEs after large reperfused ST-elevation myocardial infarction [27] .

### Study limitations

The number of patients was limited. Infarct related artery patency was not verified after baseline. Our data included patients treated with different revascularization strategies, such as thrombolysis, PCI or CABG, and cannot be generalized to solely one of these groups. Tamaki et al. have shown that acute coronary reperfusion alters kinetics of CK-MB, resulting in greater and earlier CK-MB release in the serum with equivalent infarct volume [28]. In our study, some peak CK-MB values were measured before and some after revascularization, which had influence on peak values and possibly weakened correlations to LV remodeling. Chronic phase CMR study was performed in a median of 10 (8–16) months after hospital admission; thus the period between CMRs was varied. We did not systematically evaluate the relation between troponins and remodeling, although troponins are preferred markers in detection of acute MI according to current guidelines [10], while CK-MB is the best alternative. Follow-up data was collected post-hoc and the number of MACEs was limited. We did not assess microvascular obstruction or myocardial salvage area which both omit prognostic value after MI [29].

## Conclusions

Peak CK-MB has a strong association with chronic scar size and WMAi after revascularized non-transmural MI. Considerable infarct resorption happens after the first-month recovery phase, independently of infarct size, and LV mass resorption is related to age, being more common in younger patients. At the era of increasing imaging modalities and costs, peak CK-MB provides robust estimation of infarct size, predicting chronic local and global LV function after revascularized MI.

## Abbreviations

CABG: Coronary artery bypass craft
CI: Confidence interval
CK-MB: Creatine kinase MB
CMR: Cardiovascular magnetic resonance
EDV: End-diastolic volume
EF: Ejection fraction
ESV: End-systolic volume
FLASH: Fast-low angle shot
HR: Hazard ratio
IQR: Interquartile range
LGE: Late gadolinium enhancement
LV: Left ventricular
MACE: Major adverse cardiac event
MI: Myocardial infarction
NSTEMI: Non-ST elevation myocardial infarction
PCI: Percutaneous coronary intervention
SSFP: Steady-state free-precession
STEMI: ST-elevation myocardial infarction
WMAi: Wall motion abnormality index

## References

[1] Eaton LW, Weiss JL, Bulkley BH, Garrison JB, Weisfeldt ML (1979). Regional cardiac dilatation after acute myocardial infarction: recognition by two-dimensional echocardiography. N Engl J Med.

[2] Pfeffer MA, Braunwald E (1990). Ventricular remodeling after myocardial infarction. Experimental observations and clinical implications. Circulation.

[3] St John Sutton M, Pfeffer MA, Plappert T, Rouleau JL, Moye LA, Dagenais GR, Lamas GA, Klein M, Sussex B, Goldman S (1994). Quantitative two-dimensional echocardiographic measurements are major predictors of adverse cardiovascular events after acute myocardial infarction. The protective effects of captopril. Circulation.

[4] Bolognese L, Neskovic AN, Parodi G, Cerisano G, Buonamici P, Santoro GM, Antoniucci D (2002). Left ventricular remodeling after primary coronary angioplasty: patterns of left ventricular dilation and long-term prognostic implications. Circulation.

[5] Sutton MG, Sharpe N (2000). Left ventricular remodeling after myocardial infarction: pathophysiology and therapy. Circulation.

[6] Konstam MA, Kramer DG, Patel AR, Maron MS, Udelson JE (2011). Left ventricular remodeling in heart failure: current concepts in clinical significance and assessment. JACC Cardiovasc Imaging.

[7] Gaudron P, Eilles C, Kugler I, Ertl G (1993). Progressive left ventricular dysfunction and remodeling after myocardial infarction. Potential mechanisms and early predictors. Circulation.

[8] Wu E, Ortiz JT, Tejedor P, Lee DC, Bucciarelli-Ducci C, Kansal P, Carr JC, Holly TA, Lloyd-Jones D, Klocke FJ, Bonow RO (2008). Infarct size by contrast enhanced cardiac magnetic resonance is a stronger predictor of outcomes than left ventricular ejection fraction or end-systolic volume index: prospective cohort study. Heart.

[9] Pennell DJ (2010). Cardiovascular magnetic resonance. Circulation.

[10] Thygesen K, Alpert JS, Jaffe AS, Simoons ML, Chaitman BR, White HD, Joint ESC/ACCF/AHA/WHF Task Force for the Universal Definition of Myocardial Infarction, Katus HA, Lindahl B, Morrow DA, Clemmensen PM, Johanson P, Hod H, Underwood R, Bax JJ, Bonow RO, Pinto F, Gibbons RJ, Fox KA, Atar D, Newby LK, Galvani M, Hamm CW, Uretsky BF, Steg PG, Wijns W, Bassand JP, Menasche P, Ravkilde J, Ohman EM, Antman EM, Wallentin LC, Armstrong PW, Simoons ML, Januzzi JL, Nieminen MS, Gheorghiade M, Filippatos G, Luepker RV, Fortmann SP, Rosamond WD, Levy D, Wood D, Smith SC, Hu D, Lopez-Sendon JL, Robertson RM, Weaver D, Tendera M, Bove AA, Parkhomenko AN, Vasilieva EJ, Mendis S: Third universal definition of myocardial infarction. Circulation 2012, 126(16):2020-2035. 10.1161/CIR.0b013e31826e1058 22923432

[11] Chia S, Senatore F, Raffel OC, Lee H, Wackers FJ, Jang IK (2008). Utility of cardiac biomarkers in predicting infarct size, left ventricular function, and clinical outcome after primary percutaneous coronary intervention for ST-segment elevation myocardial infarction. JACC Cardiovasc Interv.

[12] Kylmala MM, Konttila T, Vesterinen P, Kivisto SM, Lauerma K, Lindholm M, Vaananen H, Stenroos M, Nieminen MS, Hanninen H, Toivonen L (2015). Assessment of myocardial infarct size with body surface potential mapping: validation against contrast-enhanced cardiac magnetic resonance imaging. Ann Noninvasive Electrocardiol.

[13] Cerqueira MD, Weissman NJ, Dilsizian V, Jacobs AK, Kaul S, Laskey WK, Pennell DJ, Rumberger JA, Ryan T, Verani MS, American Heart Association Writing Group on Myocardial Segmentation and Registration for Cardiac Imaging (2002). Standardized myocardial segmentation and nomenclature for tomographic imaging of the heart. A statement for healthcare professionals from the cardiac imaging Committee of the Council on clinical cardiology of the American Heart Association. Circulation.

[14] McAlindon E, Pufulete M, Lawton C, Angelini GD, Bucciarelli-Ducci C (2015). Quantification of infarct size and myocardium at risk: evaluation of different techniques and its implications. Eur Heart J Cardiovasc Imaging.

[15] Kim RJ, Wu E, Rafael A, Chen E, Parker MA, Simonetti O, Klocke FJ, Bonow RO, Judd RM (2000). The use of contrast-enhanced magnetic resonance imaging to identify reversible myocardial dysfunction. N Engl J Med.

[16] Hackel DB, Reimer KA, Ideker RE, Mikat EM, Hartwell TD, Parker CB, Braunwald EB, Buja M, Gold HK, Jaffe AS (1984). Comparison of enzymatic and anatomic estimates of myocardial infarct size in man. Circulation.

[17] Hedstrom E, Astrom-Olsson K, Ohlin H, Frogner F, Carlsson M, Billgren T, Jovinge S, Cain P, Wagner GS, Arheden H (2007). Peak CKMB and cTnT accurately estimates myocardial infarct size after reperfusion. Scand Cardiovasc J.

[18] Rakowski T, Dziewierz A, Legutko J, Kleczynski P, Brzozowska-Czarnek A, Siudak Z, Urbanik A, Dubiel JS, Dudek D (2014). Creatine kinase-MB assessed in patients with acute myocardial infarction correlates with cardiac magnetic resonance infarct size at 6-month follow up. Hell J Cardiol.

[19] Dohi T, Maehara A, Brener SJ, Genereux P, Gershlick AH, Mehran R, Gibson CM, Mintz GS, Stone GW (2015). Utility of peak creatine kinase-MB measurements in predicting myocardial infarct size, left ventricular dysfunction, and outcome after first anterior wall acute myocardial infarction (from the INFUSE-AMI trial). Am J Cardiol.

[20] Choi KM, Kim RJ, Gubernikoff G, Vargas JD, Parker M, Judd RM (2001). Transmural extent of acute myocardial infarction predicts long-term improvement in contractile function. Circulation.

[21] Rodriguez-Palomares JF, Ortiz-Perez JT, Lee DC, Bucciarelli-Ducci C, Tejedor P, Bonow RO, Wu E: Time elapsed after contrast injection is crucial to determine infarct transmurality and myocardial functional recovery after an acute myocardial infarction. J Cardiovasc Magn Reson 2015, 17:43–015–0139-8. 10.1186/s12968-015-0139-8 PMC4449586 26024662

[22] Pokorney SD, Rodriguez JF, Ortiz JT, Lee DC, Bonow RO, Wu E: Infarct healing is a dynamic process following acute myocardial infarction. J Cardiovasc Magn Reson 2012, 14:62-429X-14-62. 10.1186/1532-429X-14-62 PMC3443460 22937750

[23] Lund GK, Stork A, Muellerleile K, Barmeyer AA, Bansmann MP, Knefel M, Schlichting U, Muller M, Verde PE, Adam G, Meinertz T, Saeed M (2007). Prediction of left ventricular remodeling and analysis of infarct resorption in patients with reperfused myocardial infarcts by using contrast-enhanced MR imaging. Radiology.

[24] Burns RJ, Gibbons RJ, Yi Q, Roberts RS, Miller TD, Schaer GL, Anderson JL, Yusuf S, CORE study investigators (2002). The relationships of left ventricular ejection fraction, end-systolic volume index and infarct size to six-month mortality after hospital discharge following myocardial infarction treated by thrombolysis. J Am Coll Cardiol.

[25] Palazzuoli A, Beltrami M, Gennari L, Dastidar AG, Nuti R, McAlindon E, Angelini GD, Bucciarelli-Ducci C (2015). The impact of infarct size on regional and global left ventricular systolic function: a cardiac magnetic resonance imaging study. Int J Cardiovasc Imaging.

[26] Shih H, Lee B, Lee RJ, Boyle AJ (2011). The aging heart and post-infarction left ventricular remodeling. J Am Coll Cardiol.

[27] Bodi V, Sanchis J, Nunez J, Mainar L, Lopez-Lereu MP, Monmeneu JV, Rumiz E, Chaustre F, Trapero I, Husser O, Forteza MJ, Chorro FJ, Llacer A (2009). Prognostic value of a comprehensive cardiac magnetic resonance assessment soon after a first ST-segment elevation myocardial infarction. JACC Cardiovasc Imaging.

[28] Tamaki S, Murakami T, Kadota K, Kambara H, Yui Y, Nakajima H, Suzuki Y, Nohara R, Takatsu Y, Kawai C, Tamaki N, Mukai T, Torizuka K (1983). Effects of coronary artery reperfusion on relation between creatine kinase-MB release and infarct size estimated by myocardial emission tomography with thallium-201 in man. J Am Coll Cardiol.

[29] Eitel I, de Waha S, Wohrle J, Fuernau G, Lurz P, Pauschinger M, Desch S, Schuler G, Thiele H (2014). Comprehensive prognosis assessment by CMR imaging after ST-segment elevation myocardial infarction. J Am Coll Cardiol.

